# Microglia PKM2 Mediates Neuroinflammation and Neuron Loss in Mice Epilepsy through the Astrocyte C3-Neuron C3R Signaling Pathway

**DOI:** 10.3390/brainsci13020262

**Published:** 2023-02-03

**Authors:** Xinlin Li, Rong Zhou, Hui Peng, Jing Peng, Qiaoling Li, Meng Mei

**Affiliations:** 1 Department of Pharmacy, Wuhan Children’s Hospital (Wuhan Maternal and Child Healthcare Hospital), Tongji Medical College, Huazhong University of Science and Technology, Wuhan 430015, China; 2 Institute of Maternal and Child Health, Wuhan Children’s Hospital (Wuhan Maternal and Child Healthcare Hospital), Tongji Medical College, Huazhong University of Science and Technology, Wuhan 430015, China

**Keywords:** epilepsy, neural network, neuroinflammation, PKM2, C3

## Abstract

Epilepsy is a neurological disease and approximately 30% of patients have failed to respond to current anti-epilepsy drugs. The neuroinflammation mechanism has raised increasing concerns and been regarded as the novel treatment strategy in epilepsy, but the target molecules require further research. Pyruvate kinase isoform 2 (PKM2) is well studied in peripheral inflammation, but its role in epilepsy neuroinflammation remains unclear. We knocked down microglia PKM2 in the hippocampus using a stereotaxic adeno-associated virus (AAV) microinjection and established a pilocarpine-induced status epilepticus (PISE) model. Racine score was used to evaluate the seizure grade. Next, we used WB, Multiplex tyramide signal amplification (TSA) staining and other methods to determine neuroinflammation and the complement component 3 (C3)–C3aR interaction in primary microglia. Results showed that microglia PKM2 knockdown reduced epilepsy grade and rescued neuron loss. Mechanistically, PKM2 knockdown inhibited microglia activation and inflammation factor secretion through suppressing p65 expression and phosphorylation. The reduced microglia C1q, TNF-α, and IL-1α were responsible for the decreased astrocyte C3 expression and the following neuron damage caused by the C3–C3aR interaction. Our data suggest that microglia PKM2 inhibition ameliorates neuroinflammation and neuron loss through C3-C3aR interaction in epilepsy, which provides an attractive target for the intervention of damaged neuron-glia crosstalk in epilepsy.

## 1. Introduction

Epilepsy, one of the most common neurologic diseases, is characterized by spontaneous recurrent seizures resulting from hyperexcitability and hypersynchrony of brain networks [1,2]. More than 70 million people worldwide suffer from epilepsy, and a small group of them do not respond to the currently available anti-epileptic drugs targeting at modulating voltage-gated ion channels, enhancing GABA-mediated inhibition, regulating synaptic release, inhibiting glia gap junction and synaptic excitation [3,4,5]. Accumulating evidence from experimental epilepsy models mirrored by activated glia and increased inflammation cytokines production indicate that neuroinflammation participates in the pathological process of epilepsy, and therapies targeting at neuroinflammatory pathways have vast potential [6,7,8,9,10,11]. 

Complement is part of the innate immune system, but its functions are far beyond pathogen immunosurveillance. A dysregulated complement system causes numerous disorders including inflammation-related diseases and nervous system diseases. In the brain, C3 is a vital complement component and is mainly expressed in the astrocyte. It is induced by activated microglia-released inflammation mediators. Aberrant C3 expression is detected under neuroinflammation conditions and compromises neuron migration and synapse shaping [12,13,14,15,16]. In addition, targeting the suppression of C3 excessive activation-caused neuron lesions alleviates the progression of diseases such as Parkinson’s disease [17], Alzheimer’s disease [12], stroke [18], depression [19] and epilepsy [20]. 

Pyruvate kinase, the rate-limiting enzyme of glycolysis, catalyzes phosphoenolpyruvate to pyruvate. The isoforms of PK differ depend on cell types. Remarkably, PKM2 is allosterically regulated by numerous allosteric agents and post-translational modifications [21,22,23,24,25,26]. Tetrameric PKM2 exerts cytosolic functions as the glycolytic enzyme while dimeric PKM2 possesses nuclear functions as a transcriptional coactivator and protein kinase [27,28,29,30]. Studies have demonstrated that PKM2 plays an essential role in inflammatory disorders through its canonical enzymatic function and noncanonical function. Daolin Tang et al. reported that PKM2 participates in sepsis through interacting with transcription factor HIF-1α and PKM2-depended aerobic glycolysis in macrophages thus promoting inflammasome activation and inflammatory cytokine release [31,32]. Moreover, the protein kinase activity of nuclear PKM2 that phosphorylates STAT3 followed by boosting inflammation production is verified in the macrophages of coronary artery disease [33], the CD4+ T cell of arthritis [34] and the Th17 cells of experimental autoimmune encephalomyelitis [35]. Although PKM2 has been well studied in peripheral inflammation, researchers have begun to focus on brain PKM2 only in recent years. Still, the role of PKM2 in epilepsy has not been elucidated. Especially, the PKM2-triggered microglia/astrocyte/neuron network injury mechanism in epilepsy remains to be clarified.

The aim of this study is to investigate the role and mechanism of microglia PKM2 in PISE mice. We found that microglia PKM2 knockdown relieved the incidence and seizure grades of PISE mice. Mechanistically, PKM2-induced microglia inflammation mediators complement component 1q (C1q), tumor necrosis factor-alpha (TNF-α) and interleukin-1 alpha (IL-1α) secretion through activating nuclear factor-kappa B (NF-κB), thereby promoting astrocyte C3 expression. The enhanced C3 then acts on neuron C3aR and causes neuron damage. These findings uncover the mechanism of microglia PKM2/astrocyte C3/neuron C3aR crosstalk in the progression of epilepsy.

## 2. Materials and Methods

### 2.1. Animals 

All mice care and use in the study were approved by the Institutional Animal Care and Use Committee, Huazhong University of Science and Technology. All animal procedures were carried out in compliance with the guidelines set by Huazhong University of Science and Technology (IACUC No.2861). C57BL/6J (male, 8-week-old) mice purchased from Gempharmatech Co., Ltd. were housed in an SPF environment with the same dark/light cycle (12:12), room temperature at 22–25 °C and humidity at 55–60%. All mice had ad libitum access to regular chow diet and clean water.

### 2.2. Stereotaxic AAV Microinjection

Mice were anaesthetized, fixed on a stereotaxic apparatus and sterilized with povidone iodine. After shaving the head and drilling the skull, a total of 2 × 10^9^ AAV-F4/80-NC or AAV-F4/80-PKM2 KD virus particles (Hanbio, Shanghai, China) were bilaterally microinjected into hippocampus with the coordinates of anteroposterior: −2.0 mm, mediolateral: ±1.5 mm, dorsoventral: −2 mm at a rate of 0.25 μL/min. Nineteen mice were injected with AAV-F4/80-NC virus and thirty mice were injected with AAV-F4/80-PKM2 KD virus. Three weeks later, the mice were sacrificed to assess the infection efficiency of AAV-F4/80-PKM2 KD and then subjected to the PISE model. 

### 2.3. PISE Mouse Model

All mice were intraperitoneally injected with atropine (1 mg/kg, Anhui Changjiang Pharmaceutical, Wuhu, China) to limit the peripheral effects of pilocarpine. After thirty minutes, mice in PISE groups were administrated with pilocarpine (280 mg/kg, i.p., HY-B0726, MedChemExpress, NJ, USA) while in CTL groups were treated with the equal volume of saline. Seizure grade was assessed according to the Racine scale: stage1, mouth and facial movements; stage 2, head nodding; stage 3, forelimb clonus; stage 4, rearing; and stage 5, rearing and falling [36]. Only those who attained stage 4 or 5 seizures were defined as successful PISE model. The status epilepticus was terminated using diazepam (10 mg/kg, i.p., Secondly Factory of Hainan Pharmaceutical Factory Co., Ltd., Hainan, China) in all mice 2 h later. After modeling, all mice were additionally fed with sliced peeled apples and kept warm. There were 10 mice in the AAV-NC-CTL group, 9 mice in the AAV-NC-PISE group, 14 mice in the AAV-PKM2 KD-CTL group and 16 mice in the AAV-PKM2 KD-PISE group. After being sacrificed and processed, nissl and NeuN staining were used to observe neuronal death, Iba1 staining was used to assess the activation of microglia, ionized calcium binding adapter molecule 1 (Iba1)/p-p65 double staining was used to evaluate NF-kB activation and glial fibrillary acidic protein (GFAP)/C3 double staining was used to measure astrocyte C3 expression.

### 2.4. Primary Cell Culture, Transfection and Treatment

Primary astrocytes were extracted from the brain tissue of neonatal C57BL/6J mice aged 1–3 days. The separated tissues with meninges and blood vessels removed were digested with 0.25% trypsin (25200056, Gibco, CA, USA) for 3–5 min under room temperature and terminated with Fetal Bovine Serum (FBS, 10091148, Gibco, CA, USA). Then the suspensions were filtered with 40 μm filter and centrifuged at 1000 rpm for 5 min. Dulbecco’s Modified Eagle Medium (DMEM, 12100046, Gibco, CA, USA) supplemented with 10% FBS and 1% penicillin-streptomycin (15140122, Gibco, CA, USA) was used to resuspend the cell pellet and plant it in the culture dish. The medium was replaced with fresh medium 12–18 h later and then every 3 days. The second-generation primary astrocyte could be used for the follow-up experiments.

Primary microglia were extracted from brain tissue of neonatal C57BL/6J mice aged 1–3 days. The separated tissues with meninges and blood vessels removed were digested with 0.25% trypsin and terminated with FBS. After being filtered, centrifuged and resuspended, the cell suspension was seeded into a T75 cell culture flask precoated with poly-D-lysine (P0296, Sigma, St Louis, MO, USA) in DMEM/F12 medium (11330032, Gibco, CA, USA) supplemented with 10% FBS and 1% penicillin-streptomycin. The medium was changed every 3 days. After 12–14 days, the microglia were isolated via gently shaking the T75 flasks, collected via centrifugation and seeded in a culture dish.

Primary neurons were extracted from the brain tissues of C57BL/6J foetal (E14–16). The separated tissues with meninges and blood vessels removed were digested with 0.025% trypsin for 30 min at 37 °C and terminated with FBS. Then the suspension was filtered with 40 μm filter and centrifuged at 1000 rpm for 5 min. The cell precipitates were resuspended with neurobasal medium (21103049, Gibco, CA, USA) supplemented with 1% penicillin-streptomycin, 2% B-27 (17504044, Gibco, CA, USA) and 0.5 mM L-glutamine (25030081, Gibco, CA, USA) and planted in poly-D-lysine precoated plates. The medium was half changed every 3.5 days. The primary neuron could be used for the follow-up experiments after 7 days.

Mouse PKM1 siRNA (5′-GUGCGAGCCUCCAGUCACUTT-3′, Tsingke, Beijing, China), mouse PKM2 siRNA (5′-GGCAGAGGCUGCCAUCUACTT-3′, Tsingke, Beijing, China) and NC siRNA (Tsingke, Beijing, China) were transfected into primary microglia using Lipofectamine™ 3000 (L3000015, Invitrogen, CA, USA) according to the manufacturer’s protocol.

Primary microglia pretreated with JSH-23 (JSH, 10 μM, HY-13982, MedChemExpress, NJ, USA) or solvent control dimethylsulfoxide (DMSO) or transfected with PKM2 siRNA or NC siRNA were treated with Lipopolysaccharide (LPS, 100 ng/mL, L4516, Sigma, St Louis, MO, USA) for 24 h. Primary astrocytes were treated with microglia conditioned medium (MCM) mixed with fresh DMEM at a ratio of 1:2 for 24 h. Primary neurons were treated with C3a receptor antagonist (C3aRA, 1 μM, HY-101502A, MedChemExpress, NJ, USA) or solvent control DMSO for 1 h followed by astrocyte conditioned medium (ACM) mixed with fresh neurobasal medium at a ratio of 1:2 for 24 h.

### 2.5. Immunofluorescence (IF) Analysis

Mice were anaesthetized and perfused with phosphate buffered saline (PBS). Then the brain tissues were obtained and fixed with 4% paraformaldehyde at 4 °C prior to the dehydration with 20% sucrose-PBS solution for 3 days and 30% sucrose-PBS solution for 3 days at 4 °C separately. The brain tissues were embedded with OCT glue and cut into continuous frozen slices with a thickness of 25 μm. Slices were permeabilized and blocked with 0.3% Triton X-100/5% BSA in PBS for 1.5 h at room temperature. Then the slices were incubated with NeuN (24307, Cell Signaling Technology, Danvers, MA, USA), Iba1 (17198, Cell Signaling Technology, Danvers, MA, USA), GFAP (MAB360, Millipore, MA, USA) or C3 (PA5-21349, Invitrogen, CA, USA) primary antibody at 4 °C overnight. After being washed with PBS, the second antibody anti-rabbit IgG (H + L), F(ab’)2 Fragment (Alexa Fluor^®^ 488 Conjugate) (4412, Cell Signaling Technology, Danvers, MA, USA) or anti-mouse IgG (H + L), F(ab’)2 Fragment (Alexa Fluor^®^ 555 Conjugate) (4409, Cell Signaling Technology, Danvers, MA, USA) was added and the slices were observed under a fluorescence microscope or a confocal microscope and analyzed using Image J software.

As to primary cell IF analysis, briefly, primary cells were rinsed with PBS, fixed with 4% paraformaldehyde at room temperature for 20 min, blocked with 0.3% Triton X-100/5% BSA in PBS for 1.5 h at room temperature and incubated with C3, microtubule-associated protein 2 (MAP2, 17490-1-AP, Proteintech, Chicago, IL, USA) or GFAP primary antibody. After being washed with PBS, the cells were incubated with anti-mouse IgG (H + L), F(ab’)2 Fragment (Alexa Fluor^®^ 555 Conjugate) or anti-rabbit IgG (H + L), F(ab’)2 Fragment (Alexa Fluor^®^ 488 Conjugate). To assess the average neurite length, 50 neurites of each field were measured and 5 fields of each group were counted. The neurite length was measured using Neuron J plug-in of Image J software. For C3 fluorescence intensity in GFAP-positive cell measurement, cells were observed under a fluorescence microscope and counted or analyzed using Image J software.

### 2.6. Multiplex TSA Staining

Brain slices were treated with 3% H_2_O_2_ for 15 min to quench endogenous peroxidase activity, permeabilized and blocked with 0.3% Triton X-100/5% BSA in PBS. Then, the slices were incubated with Iba1 primary antibody followed by incubating with corresponding second antibody. After being washed thoroughly, signal amplification was achieved using specific TSA dye and terminated using PBS. Then, the brain slices were subjected to eluent solution to remove unbound antibodies and reblocked. Next, the slices were stained with PKM2 (4053, Cell Signaling Technology, Danvers, MA, USA) or p-p65 ser536 (3033, Cell Signaling Technology, Danvers, MA, USA) primary antibody. The next steps were the same as before. After all antibodies were stained, the slices were finally stained with DAPI. The slices were imaged under confocal microscopy.

### 2.7. Nissl Staining

Brain slices were immersed in 0.5% cresyl violet (C-1791, Sigma, St Louis, MO, USA) at 37 °C for 20 min, washed with double distilled water and differentiated with 95% ethanol. After being dehydrated with gradient ethanol, vitrified with xylene and sealed with neutral balata, the slices were photographed and counted using Image J software (Version 1.8.0).

### 2.8. Enzyme Linked Immunosorbent Assay (ELISA)

The concentrations of supernatant C3, TNF-α, IL-1α and C1q were measured using mouse C3 ELISA Kit (E-EL-M0330c, Elabscience, Wuhan, China), mouse TNF-α ELISA Kit (EK282, Multisciences, Hangzhou, China), mouse IL-1α ELISA Kit (EK201A, Multisciences, Hangzhou, China) and mouse C1q ELISA Kit (ELK5208, ELK Biotechnology, Wuhan, China), respectively, according to the corresponding manufacturer’s protocol.

### 2.9. Hoechst33342 Staining

Primary neurons were washed thrice with ice-cold PBS followed by Hoechst33342 (B2261, Sigma, St Louis, MO, USA) staining for 30 min in the dark at room temperature. The neurons were washed with PBS, and the nuclei morphologic changes of the cells were imaged under fluorescence microscope and counted using Image J software (Version 1.8.0).

### 2.10. Real-Time PCR

Total RNA of primary microglia was extracted using FastPure^®^ Cell/Tissue Total RNA Isolation Kit V2 (RC112, Vazyme, Nanjing, China) according to the manufacturer’s protocol and reverse transcribed into cDNA using HiScript^®^ III RT SuperMix for qPCR (+gDNA wiper) (R323-01, Vazyme, Nanjing, China). The real-time PCR reaction was performed in a 10 μL reaction system including primer, cDNA, ChamQ Universal SYBR qPCR Master Mix (Q711-02, Vazyme, Nanjing, China) and DEPC water. The reaction conditions were stage 1: 95 °C 30 s, Reps 1; stage 2: 95 °C 10 s, 60 °C 30 s, Reps 40; stage 3: 95 °C 15 s, 60 °C 1 min, 95 °C 15 s, Reps 1. The primer sequences were as follows:

C3 Forward: CTGAACACAGCCAAAGATCGGA;
C3 Reverse: CCATGTTCAAGTCCTTATGGTCAG;
IL-1β Forward: AGCTTCCTTGTGCAAGTGTCTG;
IL-1β Reverse: ATCTTTTGGGGTCCGTCAACTTCA;
IL-6 Forward: GTTCCTCTCTGCAAGAGACTTC;
IL-6 Reverse: AGTCTCCTCTCCGGACTTGT;
TNF-α Forward: TGTAGCCCACGTCGTAGCA;
TNF-α Reverse: TGGGTGAGGAGCACGTAGT;
β-actin Forward: GGACTGTTACTGAGCTGCGTT;
β-actin Reverse: CGCCTTCACCGTTCCAGTT.

### 2.11. Western Blot

Proteins of primary microglia or primary astrocyte were isolated using RIPA lysis buffer containing protease (HY-K0011, MedChemExpress, NJ, USA) and phosphatase inhibitor cocktail (HY-K0022, MedChemExpress, NJ, USA). After being uniformed and denatured by boiling at 95 °C for 5 min, protein samples were loaded, processed for sodium dodecyl sulfate polyacrylamide gel electrophoresis and transferred to polyvinylidene fluoride membranes. Then the membranes were blocked with 10% skimmed milk at room temperature for 1 h followed by incubating with primary antibodies against β-actin (66009-1-Ig, Proteintech, Chicago, IL, USA), PKM2, PKM1 (15821-1-AP, Proteintech, Chicago, IL, USA), C3, p-p65 ser536, p-p65 ser468 (3039, Cell Signaling Technology, Danvers, MA, USA) and p65 (6956, Cell Signaling Technology, Danvers, MA, USA) at 4 °C overnight. After being washed with TBST, the membranes were incubated with corresponding anti-mouse (SA00001-1, Proteintech, Chicago, IL, USA) or anti-rabbit (SA00001-2, Proteintech, Chicago, IL, USA) second antibody at room temperature for 1 h and visualized by ECL chemiluminescent substrate reagent.

### 2.12. CCK8 Assay

Cell viability was measured using a Cell Counting Kit (K009, Zeta, CA, USA) in accordance with the manufacturer’s instructions. The absorbance at 450 nm was recorded using a Varioskan Flash spectral scanning multimode reader.

### 2.13. Statistical Analysis

Data are presented as mean ± SEM. SPSS software (Version 18.0) was used to analyze the data. One-way or two-way analysis of variance (ANOVA) followed by Tukey’s post hoc test and chi-square test were used for statistical significance. A * *p* < 0.05, ** *p* < 0.01, *** *p* < 0.001 was considered statistical significance. The number of replicates and repeats of individual experiments were referred in the figure legends.

## 3. Results

### 3.1. Microglia PKM2 Is Upregulated in the Hippocampus of PISE Mice Model

**Figure 1:**
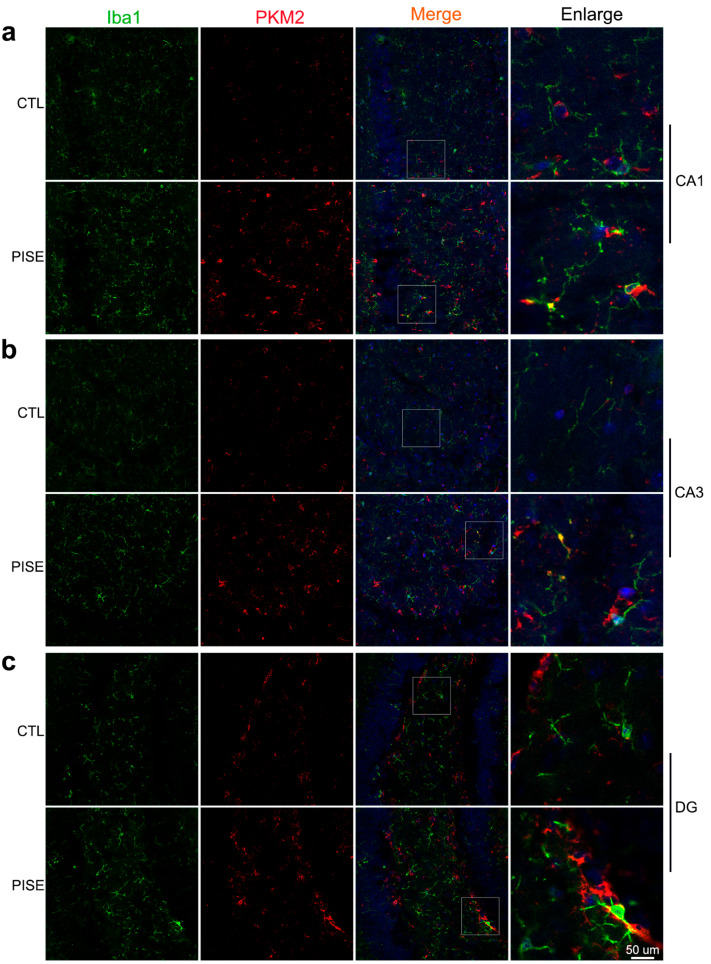
Microglia PKM2 is upregulated in the hippocampus of PISE mice model. Representative TSA staining images of PKM2 in Iba1-positive microglia in the hippocampus CA1 (**a**), CA3 (**b**), and DG region (**c**) of CTL and PISE mice. Scale bar, 50 μm. N = 3 mice per group.

To explore the role of microglia PKM2 in epilepsy, we established the PISE mice model. TSA staining showed that microglia PKM2 was upregulated in the hippocampus CA1 (Figure 1a), CA3 (Figure 1b) and DG region (Figure 1c) of PISE mice.

### 3.2. Microglia PKM2 Knockdown Alleviates PISE Seizure and Rescues Neuron Death in PISE Mice Model

**Figure 2:**
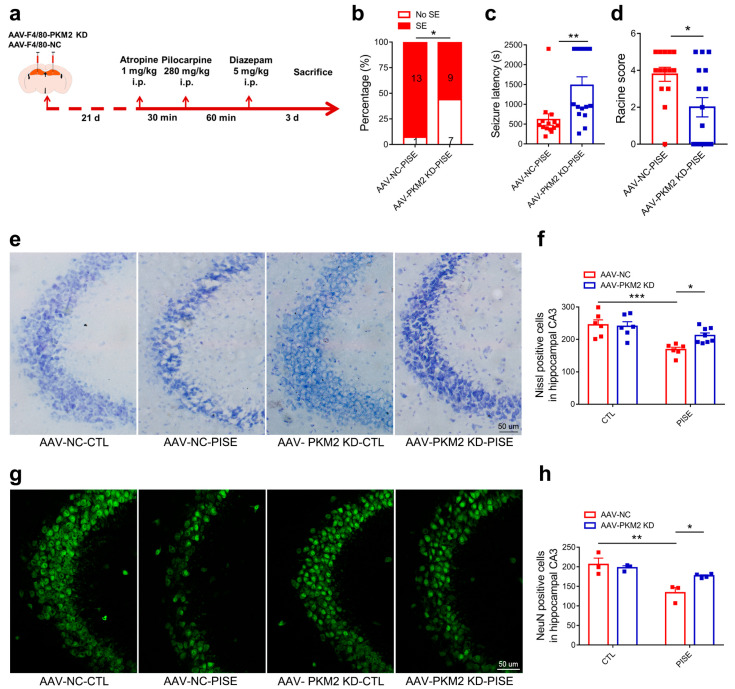
Microglia PKM2 knockdown alleviates PISE seizure and rescues neuron death in PISE mice model. (**a**) Schematic diagram showing the treatment procedure and routine of mice. The incidence ((**b**), * *p* = 0.039, compared to the AAV-NC-PISE group), latency time ((**c**), ** *p* = 0.0031, compared to the AAV-NC-PISE group) and Racine score ((**d**), * *p* = 0.012, compared to the AAV-NC-PISE group) of SE in PISE mice stereotaxically injected with AAV-F4/80-NC or AAV-F4/80-PKM2 KD. N = 14–16 mice per group. Representative nissl staining images (**e**) (Scale bar, 50 μm) and quantification of nissl-positive cell numbers ((**f**), *** *p* = 0.0007, compared to the AAV-NC-CTL group; * *p* = 0.0476, compared to the AAV-NC-PISE group) in the hippocampus CA3 region of CTL and PISE mice stereotaxically injected with AAV-F4/80-NC or AAV-F4/80-PKM2 KD. N = 6–8 mice per group. Representative IF staining images of NeuN (**g**) (Scale bar, 50 μm) and quantification of NeuN-positive cell numbers ((**h**), ** *p* = 0.0036, compared to the AAV-NC-CTL group; * *p* = 0.0476, compared to the AAV-NC-PISE group) in the hippocampus CA3 region of CTL and PISE mice stereotaxically injected with AAV-F4/80-NC or AAV-F4/80-PKM2 KD. N = 3–4 mice per group. Data are presented as mean ± SEM.

To further investigate the role of microglia PKM2 in PISE mice, microglia specific PKM2 knockdown AAV and its negative control AAV were stereotaxically injected in the hippocampus and PISE mice model was established 3 weeks later (Figure 2a). Immunostaining results of PKM2 and Iba1 showed the specificity and validity of AAV-F4/80-PKM2 KD (Figure S1a). Compared to AAV-NC-PISE group, AAV-PKM2 KD-PISE group had a lower incidence of SE, a longer latency time and a lower Racine score (Figure 2b–d). Despite the behavior changes, hippocampus neuron loss is also the typical pathological character of epilepsy. Nissl and NeuN staining of mice brain slices revealed that nissl bodies and NeuN-positive cells were significantly reduced in the hippocampus of AAV-NC-PISE group, which was alleviated in the AAV-PKM2 KD-PISE group. Moreover, microglia-specific PKM2 knockdown had no marked influence on neuron loss in CTL mice (Figure 2e–h). All the results clarified that microglia PKM2 knockdown exerted a neuroprotective effect in the PISE mice model.

### 3.3. Microglia PKM2 Knockdown Inhibits Microglia Activation via the Suppression of NF-κB Activation in PISE Mice

**Figure 3:**
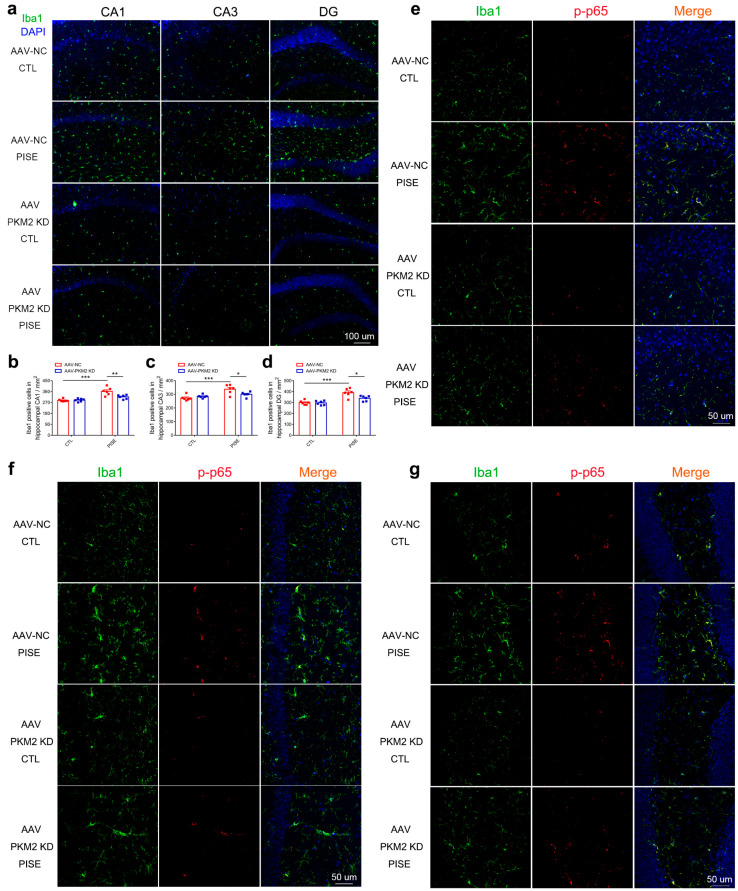
Microglia PKM2 knockdown inhibits microglia activation via the suppression of NF-κB activation in PISE mice. (**a**) Representative IF staining images of Iba1 in the hippocampus of CTL and PISE mice stereotaxically injected with AAV-F4/80-NC or AAV-F4/80-PKM2 KD (Scale bar, 100 μm). Quantification of Iba1-positive cells in the hippocampus CA1 ((**b**), *** *p* < 0.0001, compared to the AAV-NC-CTL group; ** *p* = 0.0039, compared to the AAV-NC-PISE group), CA3 ((**c**), *** *p* = 0.0003, compared to the AAV-NC-CTL group; * *p* = 0.041, compared to the AAV-NC-PISE group), and DG region ((**d**), *** *p* < 0.0001, compared to the AAV-NC-CTL group; * *p* = 0.0146, compared to the AAV-NC-PISE group) of CTL and PISE mice stereotaxically injected with AAV-F4/80-NC or AAV-F4/80-PKM2 KD. N = 6 mice per group. Representative TSA staining images of p-p65 in Iba1-positive microglia in the hippocampus CA3 (**e**), CA1 (**f**) and DG (**g**) of CTL and PISE mice stereotaxically injected with AAV-F4/80-NC or AAV-F4/80-PKM2 KD (Scale bar, 50 μm). N = 3 mice per group. Data are presented as mean ± SEM.

Neuroinflammation is a key pathogenesis and therapeutic target of epilepsy and PKM2 is involved in inflammation-related diseases. Hence, to verify whether the neuroprotective effect of microglia PKM2 knockdown is through the suppression of neuroinflammation, hippocampus Iba1 was detected. Immunostaining results showed that Iba1-positive cells were significantly increased in the hippocampus CA1, CA3 and DG region of PISE model group, while microglia PKM2 knockdown markedly inhibited the proliferation and activation (Figure 3a–d). Given that the microglia PKM2 was engaged in neuroinflammation, we further studied the mechanism of microglia PKM2 on neuroinflammation. Immunostaining results demonstrated that pilocarpine induced an increase in p-p65 expression in hippocampus microglia. However, microglia PKM2 knockdown could inhibit such an increase (Figure 3e–g), suggesting that microglia PKM2 knockdown exerted an anti-neuroinflammation effect on the PISE mice model through suppressing NF-κB activation.

### 3.4. PKM2 Knockdown Prevents C1q, TNF-α and IL-1α Secretion by Inhibiting NF-κB Activation in LPS-Activated Microglia

**Figure 4:**
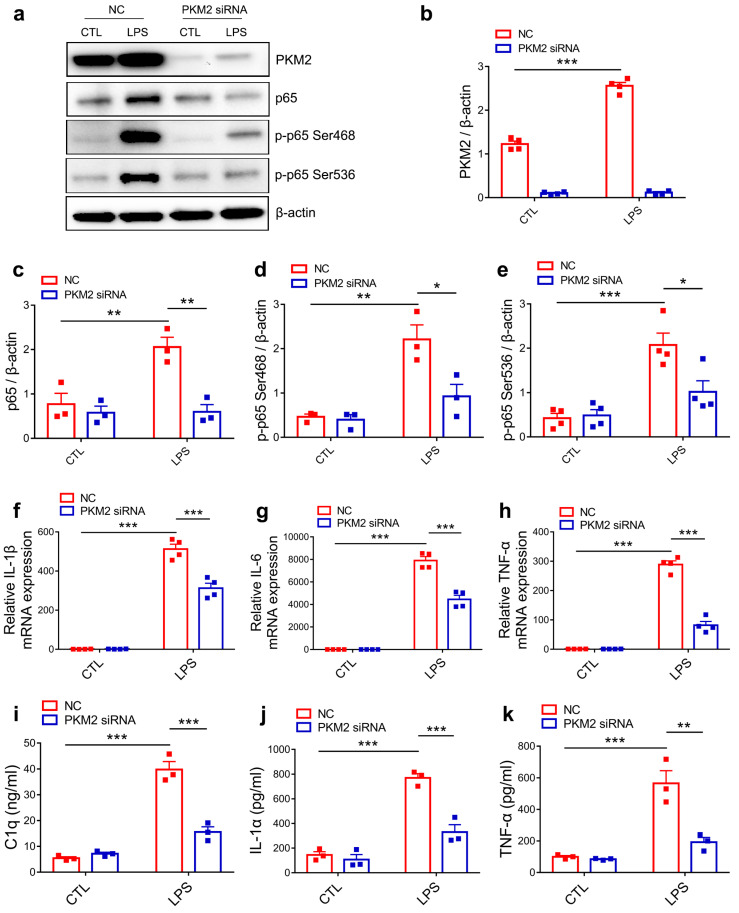
PKM2 knockdown prevents C1q, TNF-α and IL-1α secretion by inhibiting NF-κB activation in LPS-activated microglia. (**a**) Representative immunoblotting images of PKM2, p65, p-p65 Ser468 and p-p65 Ser536 in NC or PKM2 siRNA microglia treated with or without LPS. Quantification of PKM2 ((**b**),*** *p* < 0.0001, compared to the NC-CTL group), p65 ((**c**), ** *p* = 0.008, compared to the NC-CTL group; ** *p* = 0.0037, compared to the NC-LPS group), p-p65 Ser468 ((**d**), ** *p* = 0.0024, compared to the NC-CTL group; * *p* = 0.0148, compared to the NC-LPS group) and p-p65 Ser536 ((**e**), *** *p* = 0.0004, compared to the NC-CTL group; * *p* = 0.0139, compared to the NC-LPS group) in NC or PKM2 siRNA microglia treated with or without LPS. For three to four independent experiments. QPCR measurement of IL-1β ((**f**), *** *p* < 0.0001, compared to the NC-CTL group; *** *p* < 0.0001, compared to the NC-LPS group), IL-6 ((**g**), *** *p* < 0.0001, compared to the NC-CTL group; *** *p* < 0.0001, compared to NC-LPS group) and TNF-α ((**h**), *** *p* < 0.0001, compared to the NC-CTL group; *** *p* < 0.0001, compared to the NC-LPS group) mRNA expression in NC or PKM2 siRNA microglia treated with or without LPS. For four independent experiments. Quantitative of supernatant C1q ((**i**), *** *p* < 0.0001, compared to the NC-CTL group; *** *p* < 0.0001, compared to the NC-LPS group), IL-1α ((**j**), *** *p* < 0.0001, compared to the NC-CTL group; *** *p* = 0.0004, compared to the NC-LPS group) and TNF-α ((**k**), *** *p* = 0.0003, compared to the NC-CTL group; ** *p* = 0.0013, compared to the NC-LPS group) concentration in NC or PKM2 siRNA microglia treated with or without LPS. For three independent experiments. Data are presented as mean ± SEM.

Since both PKM1 and PKM2 are derived from the alternative splicing of PKM pre-mRNA and differed only by containing exon 9 (PKM1) or exon 10 (PKM2) [37], we firstly verified the specificity and validity of PKM2 siRNA. Immunoblotting results showed that PKM2 siRNA significantly decreased the protein expression of PKM2 but had no effect on the expression of PKM1 (Figure S2a–c). As in the PISE model, LPS stimuli induced an increase in PKM2 expression. To further explore the inhibitory effect of PKM2 siRNA on NF-κB activation in primary microglia, we detected the protein expression of p65, p-p65 Ser468 and p-p65 Ser536 and found that PKM2 knockdown reduced LPS-induced upregulation of p65, p-p65 Ser468 and p-p65 Ser536 protein expression (Figure 4a–e). Notably, qPCR results showed that PKM2 knockdown significantly decreased the mRNA expression of NF-κB target gene IL-1β, IL-6 and TNF-α in LPS-treated primary microglia (Figure 4f–h). These results are consistent with those in the PISE mice model, indicating that PKM2 knockdown alleviates microglia activation through inhibiting the NF-κB pathway. Since activated microglia induced astrocyte to A1 type through secreting C1q, TNF-α and IL-1α, we next determined the concentration of the cytokines released by activated microglia using ELISA. Results showed that LPS induced the increase in C1q, TNF-α and IL-1α release. However, PKM2 siRNA treatment (Figure 4i–k) as well as NF-κB inhibitor JSH group (Figure S3a–c) blocked the increase. Taken together, PKM2 knockdown reduced C1q, TNF-α and IL-1α secretion in the activated microglia through inhibiting NF-κB activation.

### 3.5. Microglia PKM2 Knockdown Attenuates Astrocyte C3 Expression

**Figure 5:**
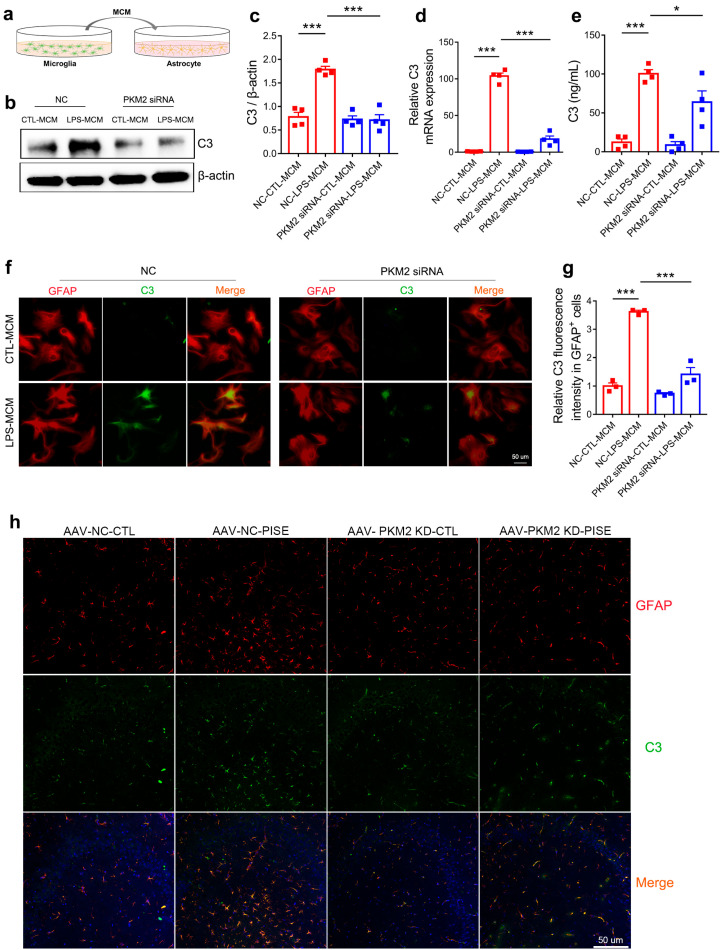
Microglia PKM2 knockdown attenuates astrocyte C3 expression. (**a**) Schematic diagram showing the treatment of MCM on astrocyte. (**b**) Representative immunoblotting images of C3 in astrocyte treated with MCM of primary microglia transfected with NC or PKM2 siRNA followed by PBS or LPS treatment groups. Quantification of C3 protein expression ((**c**), *** *p* < 0.0001, compared to the NC-CTL-MCM group; *** *p* < 0.0001, compared to the NC-LPS-MCM group), C3 mRNA expression ((**d**), *** *p* < 0.0001, compared to the NC-CTL-MCM group; *** *p* < 0.0001, compared to NC-LPS-MCM group) and supernatant C3 concentration ((**e**), *** *p* < 0.0001, compared to the NC-CTL-MCM group; * *p* = 0.0374, compared to the NC-LPS-MCM group) in astrocyte treated with MCM of primary microglia transfected with NC or PKM2 siRNA followed by PBS or LPS treatment groups. For four independent experiments. Representative IF staining images of C3 (**f**) (scale bar, 50μm) and quantification of relative C3 fluorescence intensity in GFAP-positive cells ((**g**), *** *p* < 0.0001, compared to the NC-CTL-MCM group; *** *p* < 0.0001, compared to the NC-LPS-MCM group) in astrocyte treated with MCM of primary microglia transfected with NC or PKM2 siRNA followed by PBS or LPS treatment groups. For three independent experiments. (**h**) Representative IF staining images of C3 in GFAP-positive astrocyte in the hippocampus of CTL and PISE mice stereotaxically injected with AAV-F4/80-NC or AAV-F4/80-PKM2 KD (Scale bar, 50 μm). N = 3 mice per group. Data are presented as mean ± SEM.

In the brain, C3 is mainly produced by activated astrocyte and regarded as a marker of neurotoxic reactive astrocyte [13,38]. We next wanted to investigate the ability of microglia PKM2 knockdown on astrocyte C3 expression. MCM of primary microglia transfected with NC or PKM2 siRNA followed by PBS or LPS treatment groups was collected and applied to primary astrocyte (Figure 5a). NC-LPS-MCM conducted a marked increase in astrocyte C3 protein and mRNA expression. However, MCM from the PKM2 siRNA combined LPS group could inhibit such upregulation (Figure 5b–d). As to C3 release, ELISA measurement on primary astrocyte supernatant showed that NC-LPS-MCM enhanced C3 release but could be prevented by PKM2 siRNA-LPS-MCM treatment (Figure 5e). Furthermore, immunostaining of GFAP and C3 in primary astrocyte also indicated the inhibitory ability of PKM2 siRNA-LPS-MCM on C3 expression compared with NC-LPS-MCM (Figure 5f,g). To further illustrate whether microglia PKM2 knockdown attenuated astrocyte C3 expression through microglia NF-κB pathway, we collected MCM of primary microglia pretreated with JSH followed by PBS or LPS treatment groups and applied to astrocyte. Results showed that JSH-LPS-MCM reduced astrocyte C3 protein expression and supernatant C3 release when compared to DMSO-LPS-MCM (Figure S4a–c). Furthermore, to study the in vivo role of microglia PKM2 on C3 expression in PISE mice model, immunostaining of GFAP and C3 in hippocampus was conducted. Confocal images revealed that astrocyte C3 was increased in PISE mice model and microglia PKM2 knockdown suppressed the upregulation (Figure 5h). In general, both the in vivo and in vitro results illustrated that microglia PKM2 knockdown attenuated C3 expression through inhibiting microglia C1q, TNF-α and IL-1α secretion.

### 3.6. Microglia PKM2 Knockdown Rescues Neurons via Microglia NF-κB/astrocyte C3/neuron C3aR Interaction

**Figure 6:**
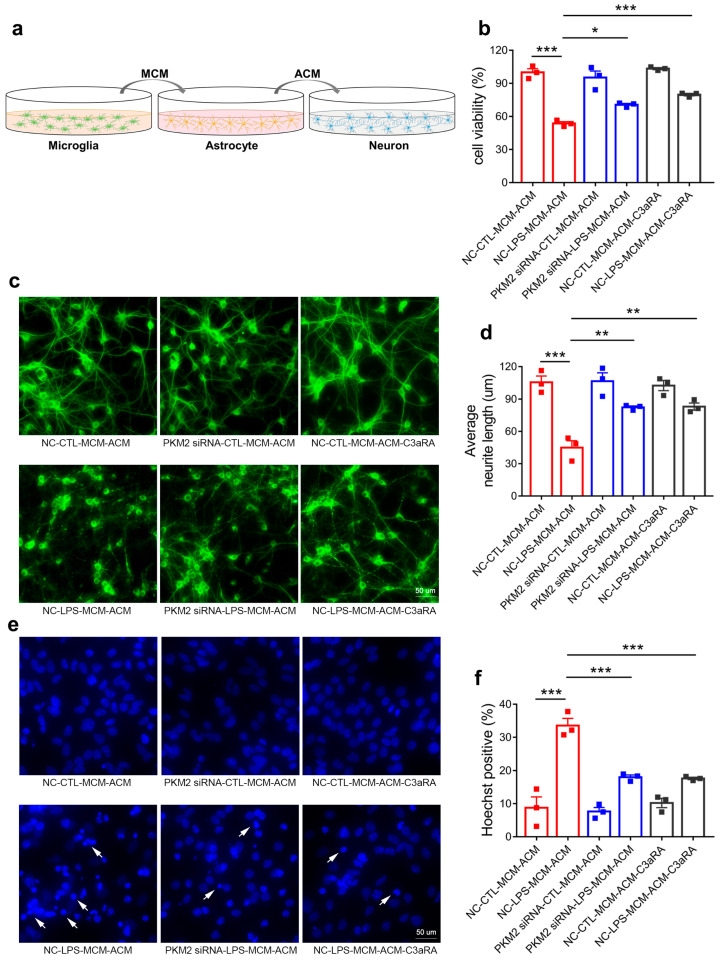
Microglia PKM2 knockdown rescues neurons via microglia NF-κB/astrocyte C3/neuron C3aR interaction. (**a**) Schematic diagram showing the administration of MCM-treated ACM on primary neuron. Cell viability ((**b**), *** *p* < 0.0001, compared to the NC-CTL-MCM-ACM group; * *p* = 0.0143, compared to the NC-LPS-MCM-ACM group; *** *p* = 0.0004, compared to the NC-LPS-MCM-ACM group), representative MAP2 IF staining images (**c**) (Scale bar, 50 μm), average neurite length ((**d**), *** *p* < 0.0001, compared to the NC-CTL-MCM-ACM group; ** *p* = 0.0033, compared to NC-LPS-MCM-ACM group; ** *p* = 0.0028, compared to NC-LPS-MCM-ACM group), representative Hoechst33342 staining images (**e**) (Scale bar, 50 μm) and hoechst33342 positive rate ((**f**), *** *p* < 0.0001, compared to the NC-CTL-MCM-ACM group; *** *p* = 0.0005, compared to the NC-LPS-MCM-ACM group; *** *p* = 0.0004, compared to the NC-LPS-MCM-ACM group) of primary neurons applied with ACM of NC-CTL-MCM, NC-LPS-MCM, PKM2 siRNA-CTL-MCM and PKM2 siRNA-LPS-MCM-treated group or pretreated with C3aRA followed by NC-CTL-MCM-ACM and NC-LPS-MCM-ACM administration. The white arrows indicate the damaged nucleus. For three independent experiments. Data are presented as mean ± SEM.

C3 is cleaved by C3 convertases to generate peptide C3a and C3b. C3a is then released and binds to the neuron C3a receptor to modulate neuron function. To explore the ultimate effect of microglia PKM2 on the neuron, we collected the ACM of the NC-CTL-MCM, NC-LPS-MCM, PKM2 siRNA-CTL-MCM and PKM2 siRNA-LPS-MCM-treated group and then applied it to the primary neuron (Figure 6a). To test whether C3 contained in the ACM played an essential role in the primary neuron, C3aRA was used as a positive control. Primary neuron pretreated with C3aRA and then applied with ACM of NC-LPS-MCM group and NC-CTL-MCM group. The CCK8 results showed that the ACM of NC-LPS-MCM group decreased primary neuron viability while the ACM of PKM2 siRNA-LPS-MCM group, as well as the NC-LPS-MCM-ACM-C3aRA group, ameliorated the neuron lesions, again certifying the neuroprotective role of PKM2 siRNA-LPS-MCM-ACM on NC-LPS-MCM-ACM-mediated neuron damage (Figure 6b). Morphologically, the NC-LPS-MCM-ACM group caused neurite length shortening, nuclei pyknosis and chromatin condensation. However, the primary neuron treated with PKM2 siRNA-LPS-MCM-ACM led to a significant improvement of neurite and nuclei morphology as well as the NC-LPS-MCM-ACM-C3aRA group (Figure 6c–f). Taken together, the results demonstrate that microglia PKM2 knockdown rescued hippocampus neurons via the microglia NF-κB/astrocyte C3/neuron C3aR interaction.

## 4. Discussion

PKM2 has been well studied in cancer and peripheral inflammation-related diseases. Its role in peripheral immune cells has been well studied. As brain-resident immune cells [39], the role of PKM2 in microglia has not been sufficiently studied but has begun to attract researchers’ attention in recent years. Zengqiang Yuan et al. reported that PKM2 blockage ameliorated microglia activation-mediated neuroinflammation by inhibiting the glycolysis/H4K12la/PKM2 positive feedback loop, thus reduced Aβ pathology and improved the spatial learning and memory in AD model mice [40]. Dongmei Zhang et al. proposed that PKM2 directly interacted with ATF2 to bridge microglia glycolysis and neuroinflammation [41]. In addition to the effect of PKM2 on microglia-medicated neuroinflammation, PKM2 also impaired microglia synaptic pruning [42]. In this study, we firstly discovered that microglia PKM2 was upregulated in the hippocampus of PISE mice. Moreover, interrupting such an increase utilizing microglia specific PKM2 knockdown AAV ameliorated microglia activation and pathological lesion in epilepsy mice. As for the mechanism, we found that PKM2 was associated with the increased expression and phosphorylation of NF-κB p65 in microglia, thereby augmenting NF-κB transcription and contributing to the release of inflammation factors C1q, TNF-α and IL-1α. Functionally, PKM2 is the limited enzyme in glysolysis, and is considered as the coactivator of the transcription factor and the protein kinase. Whether PKM2 is involved in epilepsy through its classical role of regulating glucose metabolism and coupling gap junctions remains further research. Moreover, gap junction channel is formed by connexin 43 and connexin 30, and connexin 43 has multiple phosphorylation sites and is regulated by phosphorylation [43,44,45]. Whether PKM2 acts as the protein kinase and whether phosphorylates connexin 43 and mediates gap junction remains to be clarified. Furthermore, whether PKM2 acts as a co-activator of NF-κB to directly bind to it and mediate the transactivation needs to be investigated further.

Astrocytes possess complex functions as support cells for neurons through neuronal-astrocyte interactions including gap junction, glia-neuron lactate shuttle, etc., which affects neuron function on local and neural networks [46,47,48]. Activated microglia-secreted inflammation factors C1q, TNF-α and IL-1α act together to convert astrocytes to neurotoxic reactive astrocytes, termed A1 astrocytes [13]. A1 astrocytes participate in the onset and development of various central nervous system diseases including Parkinson’s disease, aging, depression and stroke. Hence, researchers have pointed out that inhibiting the formation of A1 astrocytes can be a useful therapy targeted at the related diseases [14,49,50,51,52,53]. Among all the complement components that participate in complement activation, C3 is the critical molecule of the process since all three initial pathways converge to C3 and the cleavage of C3 causes a cascade of downstream reaction. Therefore, C3 is considered as a significantly potential target in modifying complement-mediated disease [12,54,55,56]. Moreover, C3 is the marker of A1 astrocytes. Hence, in the present study, we studied the effect of microglia PKM2 on astrocyte C3 expression and found that MCM from microglia PKM2 knockdown decreased astrocyte C3 expression, which is consistent with the C3 expression in MCM of NF-κB inhibitor JSH-treated microglia. Moreover, microglia-specific PKM2 knockdown also reduced astrocyte C3 activation in PISE mice. These results indicate that microglia PKM2 knockdown suppressed astrocyte C3 expression via decreasing microglia C1q, TNF-α and IL-1α release caused by NF-κB activation.

The C3-C3aR pathway is implicated in a variety of central nervous system diseases via mediation of the neuron–glia network function containing synapse elimination and refinement, cognitive function, dendritic morphology, neuroinflammation and neurodegeneration [12,16,57,58,59,60]. Enhanced C3 has been detected in experimental epilepsy [20,38]. We found that treatment with ACM from NC-LPS-MCM killed the neurons, while PKM2 siRNA-LPS-MCM-ACM restored neuron activity as well as those pretreated with C3aRA. The similar effect of C3aRA and PKM2 siRNA-LPS-MCM-ACM on reducing C3–C3aR interaction verified the role of microglia PKM2 on the C3/C3aR pathway in neuroinflammation in epilepsy. However, C3aR is expressed on both microglia and neuron. Microglia C3aR has been reported to mediate immune homeostasis and microglial phagocytosis [60,61]. Another limitation of this study is that we only investigated the effects of C3 on neuron C3aR. The vicious circle of astrocyte C3/microglia C3aR communication requires further research.

## 5. Conclusions

**Figure 7:**
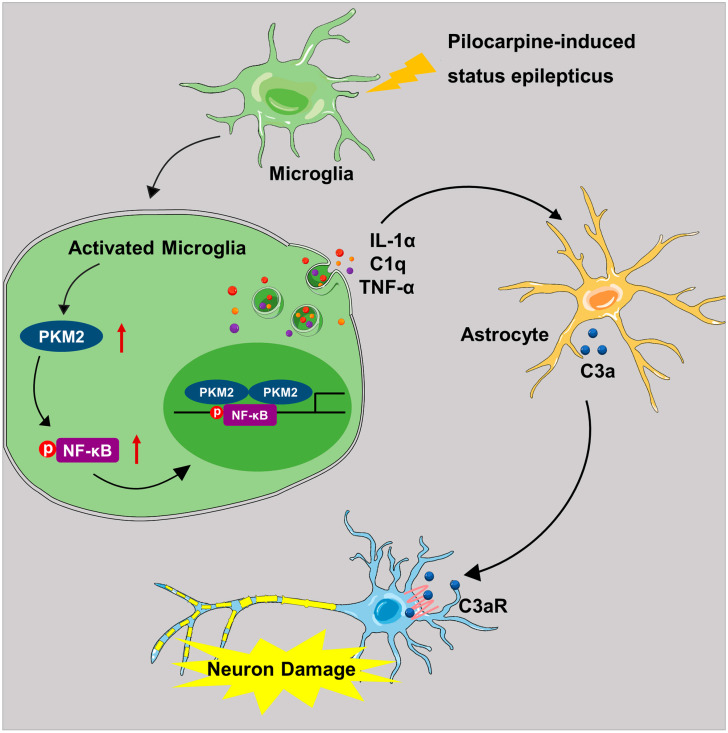
Schematic diagram illustrating the role and mechanism of microglia PKM2 in PISE mice model. In the PISE mice model, microglia PKM2 is up-regulated, thus facilitating NF-κB p65 phosphorylation. The activated microglia promote cytokines C1q, TNF-α and IL-1α secretion followed by A1-like astrocyte conversion and increased C3 expression. Then the astrocyte-secreted C3 peptide binds to neuron C3a receptor and induces neuron damage.

The present study demonstrated that microglia PKM2 was increased in the hippocampus of PISE mice model. Microglia-specific PKM2 knockdown reduced epilepsy incidence and ameliorated the pathology of PISE mice. Mechanistically, PKM2 activates microglia through elevating NF-κB expression and phosphorylation and promoting inflammation production C1q, TNF-α and IL-1α secretion, which contributes to excessive astrocyte C3 expression. Then astrocyte C3 binds to the neuron C3a receptor and induces neuron damage (Figure 7). Our results provide novel insights into the understanding of microglia PKM2/astrocyte C3/neuron C3aR network mechanism in epilepsy and preliminarily implies microglia PKM2 an attractive target for the intervention of complex cascade pathogenesis in epilepsy.

## Data Availability

Information about the experimental methods, animal model, and data used and analyzed during the current study is available from the corresponding author upon reasonable request.

## Supplementary Materials

The following supporting information can be downloaded at: https://www.mdpi.com/article/10.3390/brainsci13020262/s1, Figure S1: The knockdown efficiency of AAV-F4/80-PKM2 KD in the mice hippocampus; Figure S2: The knockdown efficiency of PKM2 siRNA in primary microglia; Figure S3: NF-κB inhibition attenuates LPS-induced C1q, TNF-α and IL-1α secretion in microglia; Figure S4: Microglia NF-κB inhibition reduces astrocyte C3 expression.

## Abbreviations

ACM: Astrocyte conditioned medium; C1q: Complement component 1q; C3: Complement component 3; C3aRA: C3a receptor antagonist; ELISA: Enzyme linked immunosorbent assay; FBS: Fetal bovine serum; GFAP: Glial fibrillary acidic protein; Iba1: Ionized calcium binding adapter molecule 1; IF: Immunofluorescence; IL-1α: Interleukin-1 alpha; IL-1β: Interleukin-1 beta; IL-6: Interleukin-6; LPS: Lipopolysaccharide; MAP2: Microtubule associated protein 2; MCM: Microglia conditioned medium; NF-κB: Nuclear factor-kappa B; PBS: Phosphate buffered saline; PISE: Pilocarpine-induced status epilepticus; PKM2: Pyruvate kinase isoform 2; TNF-α: Tumor necrosis factor alpha; TSA: tyramide signal amplification.
