# Supplementary material for: Microglia PKM2 Mediates Neuroinflammation and Neuron Loss in Mice Epilepsy through the Astrocyte C3-Neuron C3R Signaling Pathway
Source: Brain Sci. 2023 Feb 3;13(2):262. doi: 10.3390/brainsci13020262 (PMC9954168; doi:10.3390/brainsci13020262)
Supplement: Supplementary file 1 [file brainsci-13-00262-s001.zip › brainsci-2170859-supplementary.pdf]

Supplementary Figure S1

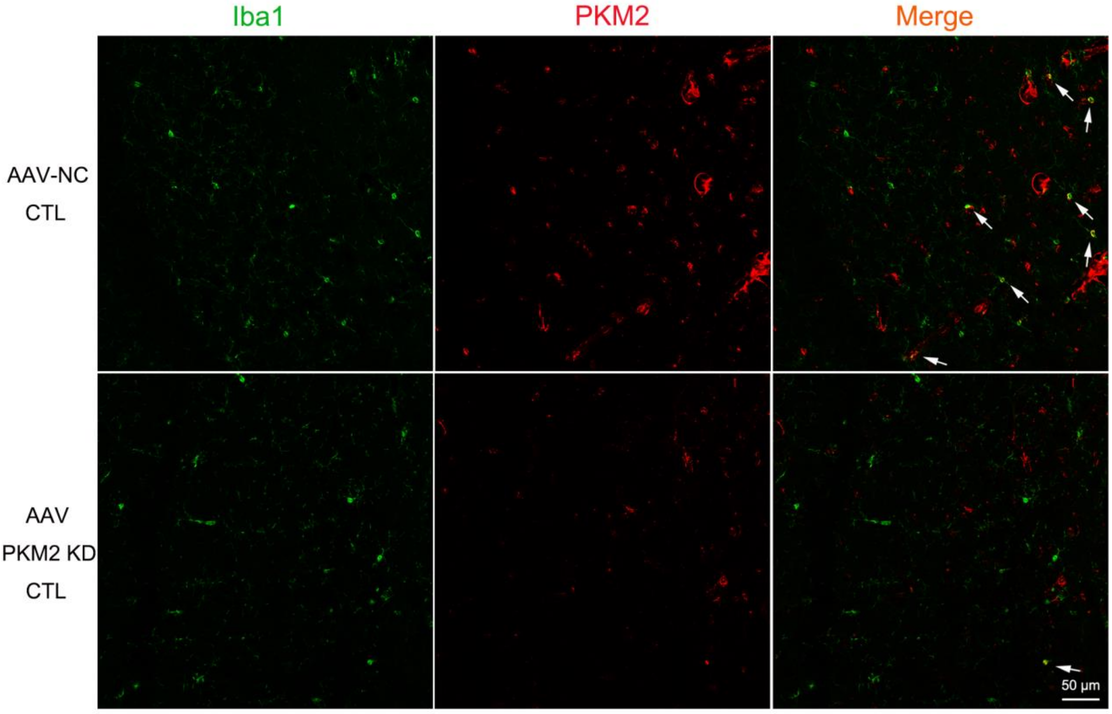

Figure S1. The knockdown efficiency of AAV-F4/80-PKM2 KD in the mice hippocampus. (**a**) Representative TSA staining images of PKM2 in Iba1-positive microglia in the hippocampus of CTL mice stereotaxically injected with AAV-F4/80-NC orAAV-F4/80-PKM2 KD. Scale bar, 50 μm. N=3 mice per group.

Supplementary Figure S2

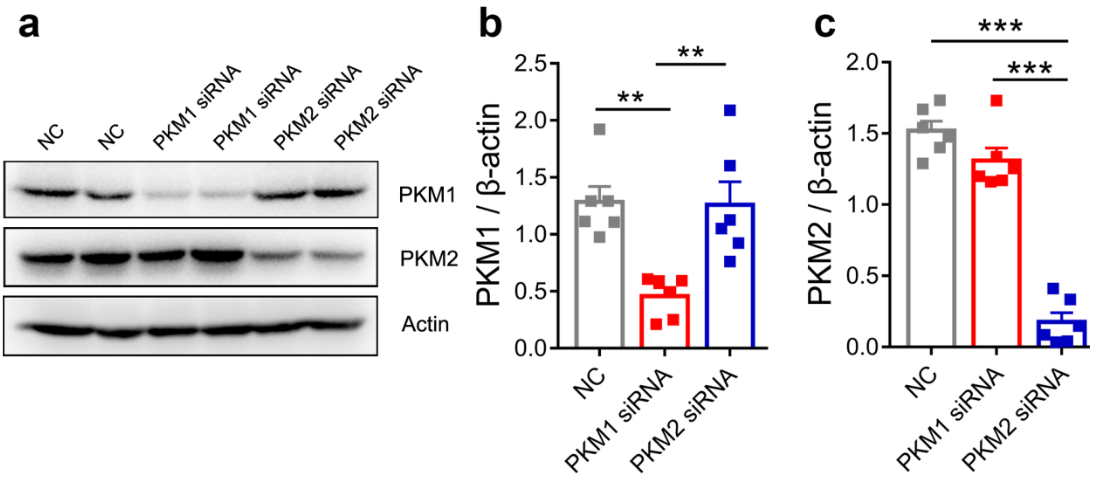

Figure S2. The knockdown efficiency of PKM2 siRNA in primary microglia. **(a)** Representative immunoblotting images of PKM1 and PKM2 in primary microglia transfected with NC siRNA, PKM1 siRNA or PKM2 siRNA. **(b)** Quantification of PKM1 protein expression in primary microglia transfected with NC siRNA, PKM1 siRNA or PKM2 siRNA**.** ** p = 0.0033, compared to the NC group; ** p = 0.0042, compared to the PKM1 siRNA group**. (c)** Quantification of PKM2 protein expression in primary microglia transfected with NC siRNA, PKM1 siRNA or PKM2 siRNA. *** p < 0.0001, compared to the NC group; *** p < 0.0001, compared to the PKM1 siRNA group. For six independent experiments. Data are presented as mean ± SEM.

Supplementary Figure S3

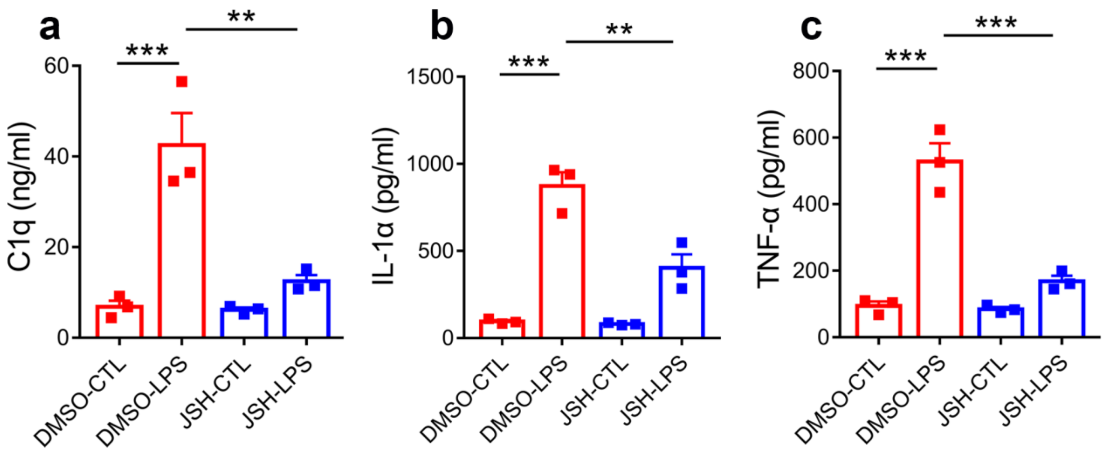

Figure S3. NF-κB inhibition attenuates LPS-induced C1q, TNF-α and IL-1α secretion in microglia. Quantitative of supernatant C1q **(a**, *** p = 0.0006, compared to the DMSO-CTL group; ** p = 0.0018, compared to the DMSO-LPS group**)**, IL-1α **(b,** *** p < 0.0001, compared to the DMSO-CTL group; ** p = 0.0015, compared to the DMSO-LPS group**)** and TNF-α **(c,** *** p < 0.0001, compared to the DMSO-CTL group; *** p = 0.0001, compared to the DMSO-LPS group**)** concentration in DMSO or JSH pretreated primary microglia accompany with or without LPS. For three independent experiments. Data are presented as mean ± SEM.

Supplementary Figure S4

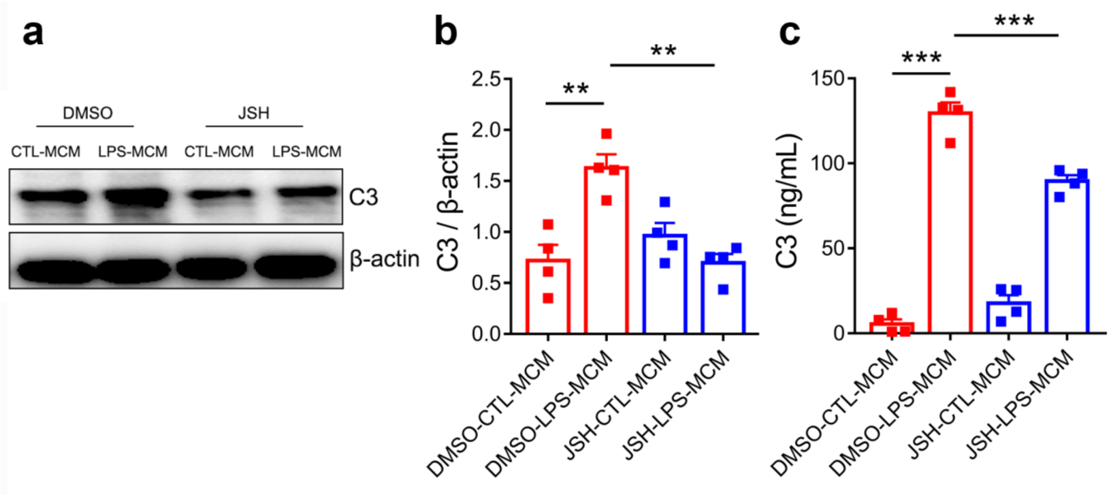

Figure S4. Microglia NF-κB inhibition reduces astrocyte C3 expression. Representative immunoblotting images (**a**) and quantification of C3 protein **(b,** **p=0.0015, compared to the DMSO-CTL-MCM group; **p=0.0012, compared to the DMSO-LPS-MCM group**)** in astrocyte treated with MCM of primary microglia pretreated with JSH or DMSO followed by PBS or LPS treatment groups. For four independent experiments. (**c**) ELISA measurement of astrocyte supernatant C3 concentration in astrocyte treated with MCM of primary microglia pretreated with JSH or DMSO followed by PBS or LPS treatment groups**;** ***p<0.0001, compared to the DMSO-CTL-MCM group; ***p=0.0002, compared to the DMSO-LPS-MCM group. For four independent experiments. Data are presented as mean ± SEM.
